# Both Enantiomers of 2-Hydroxyglutarate Modulate the Metabolism of Cultured Human Neuroblastoma Cells

**DOI:** 10.1007/s11064-024-04188-8

**Published:** 2024-06-12

**Authors:** Eduard Gondáš, Eva Baranovičová, Peter Bystrický, Jakub Šofranko, Andrea Evinová, Matúš Dohál, Zuzana Hatoková, Radovan Murín

**Affiliations:** 1 Department of Medical Biochemistry, Jessenius Faculty of Medicine in Martin, Comenius University in Bratislava, Malá hora 4D, 036 01 Martin, Slovakia; 2 Department of Pharmacology, Jessenius Faculty of Medicine in Martin, Comenius University in Bratislava, Bratislava, Slovakia; 3 Biomedical Centre Martin, Jessenius Faculty of Medicine in Martin, Comenius University in Bratislava, Bratislava, Slovakia; 4 Department of Pharmaceutical Analysis and Nuclear Pharmacy, Faculty of Pharmacy, Comenius University in Bratislava, Bratislava, Slovakia; 5 Department of Medical Biochemistry, Jessenius Faculty of Medicine in Martin, Comenius University in Bratislava, Malá hora 4D, 036 01 Martin, Slovakia

**Keywords:** 2-Hydroxyglutarate, Branched-chain Amino acid, Branched-chain keto acid, Ketone body, Neuroblastoma, Metabolism

## Abstract

Elevated levels of D-2-hydroxyglutarate (D-2HG) and L-2-hydroxyglutarate (L-2HG) in the brain are associated with various pathological conditions, potentially contributing to neurological symptoms and neurodegeneration. Previous studies on animal models have revealed their capability to interfere with several cellular processes, including mitochondrial metabolism. Both enantiomers competitively inhibit the enzymatic activity of 2-oxoglutarate-dependent dioxygenases. These enzymes also execute several signaling cascades and regulate the level of covalent modifications on nucleic acids or proteins, e.g., methylation, hydroxylation, or ubiquitination, with an effect on epigenetic regulation of gene expression, protein stability, and intracellular signaling. To investigate the potential impact of 2HG enantiomers on human neuronal cells, we utilized the SH-SY5Y human neuroblastoma cell line as a model. We employed proton nuclear magnetic resonance (^1^H-NMR) spectroscopy of culture media that provided high-resolution insights into the changes in the content of metabolites. Concurrently, we performed biochemical assays to complement the ^1^H-NMR findings and to estimate the activities of lactate and 3-hydroxybutyrate dehydrogenases. Our results reveal that both 2HG enantiomers can influence the cellular metabolism of human neuroblastoma cells on multiple levels. Specifically, both enantiomers of 2HG comparably stimulate anaerobic metabolism of glucose and inhibit the uptake of several essential amino acids from the culture media. In this respect, both 2HG enantiomers decreased the catabolism capability of cells to incorporate the leucine-derived carbon atoms into their metabolism and to generate the ketone bodies. These results provide evidence that both enantiomers of 2HG have the potential to influence the metabolic and molecular aspects of human cells. Furthermore, we may propose that increased levels of 2HG enantiomers in the brain parenchyma may alter brain metabolism features, potentially contributing to the etiology of neurological symptoms in patients.

**Supplementary Information:**

The online version contains supplementary material available at 10.1007/s11064-024-04188-8.

## Introduction

The intermediate metabolism of human cells embraces several reactions generating both enantiomers, D and L, of 2-hydroxyglutarate (2HG) [1–5]. As a cognate 2-hydroxy acid of 2-oxoglutarate (2OG), 2HG could reenter the cellular metabolism by subsequent enzymatic oxidation catalyzed by a corresponding stereospecific 2-hydroxyglutarate dehydrogenase (2HGDH). Under physiological conditions, D- and L-2HG levels remain at few micromolar levels in the human cells, cerebrospinal fluid, and blood, with the prevailing occurrence of L-2HG in brain parenchyma [3]. In addition to hypoxia, which could stimulate a transient rise in 2HG levels by altering the substrate specificity of lactate dehydrogenase and/or malate dehydrogenase [6, 7], the genetic aberrations in either of the genes coding for D-2HGDH, L-2HGDH, or either of two NADP^+^-dependent isocitrate dehydrogenase isoforms (IDH) underlie the substantial increase of 2HG levels and could lead to one of the three types of 2-hydroxyglutaric acidurias [3, 4, 7, 8]. All types of 2-hydroxyglutaric acidurias are linked with neurological symptoms and neurodegeneration, which suggest the possible detrimental effect on the metabolism of neuronal cells [3, 8].

The molecular mechanism and cellular targets of 2HG-associated neurological symptoms and brain damage are not fully elucidated yet. It has been shown, that both 2HG enantiomers might affect several cellular processes. Since, both are potent competitive inhibitors of 2OG-dependent dioxygenases [9] they can affect cellular mTOR signaling pathway [10, 11], activation of hypoxia-inducible factor (HIF) [12] and demethylation processes of both, DNA and histones, with subsequent effect on the regulation of the gene expression [5, 13, 14]. In addition, a recent study showed that 2HG could affect the cellular energetic metabolism also by inhibiting the F_O_-subunit of ATP synthase and, therefore, retarding the cellular respiration with the subsequent depletion of cellular ATP [10, 11].

Little is known about the effect of 2HG on human neuronal cells. As one of the cellular constituents of brain parenchyma, neuronal metabolism depends on the selective supply of nutrients from the bloodstream through the blood-brain barrier and intercellular cooperation with glial cells [15]. In addition to glucose, which is the main substrate for the brain energy metabolism, the brain cells can also catabolize some of the essential amino acids (EAAs) that are easily imported into the brain parenchyma and incorporated into the neuronal metabolism. Besides their universal role as the indispensable monomers for proteosynthesis, EAAs also play the roles of signaling molecules or their precursors as well as nitrogen, sulfur, or carbon atom sources in cellular brain metabolism. The raised level of 2HG inhibits the catabolism of lysine in both, mouse [16] and the patients with L-2-hydroxyglutaric aciduria [3]. Disbalance in the brain metabolism of EAAs is commonly linked with several neurological symptoms and neurodegeneration. The metabolic cooperation among the cells in the brain parenchyma requires the mutual exchange of the compounds between intracellular compartment and extracellular fluid. In a such way, 2HG could affect not only the cells of its origin, but may be distributed by extracellular fluid and might influence the metabolism of the distal cells, too. Such local producers of 2HG in brain parenchyma can be the cells affected by focal ischemia, hypoxia, glioblastomas or bearing the mutated genes of the enzymes participating on 2HG metabolism [2–5, 8].

Therefore, here we tested the possibility that increased levels of 2HG in culture media might compromise the cellular metabolism of human neural cells. As the study model of human neuronal cells, we applied human neuroblastoma cell line SH-SY5Y. Our results revealed, that both enantiomers of 2HG are metabolized by human neuroblastoma cells and the both can potently inhibit mitochondrial oxygen consumption, stimulate the anaerobic metabolism of glucose and alter the metabolism of EAAs, especially branched-chain amino acids (BCAAs). Such metabolic changes in cellular metabolism of human neuroblastoma cells provide a hint, that 2HG might exert its pathological effect by modulation of the cellular oxidative metabolism and metabolism of EAAs, and in such way to contribute to etiopathogenesis of the neurological symptoms and neurodegeneration linked with 2HG-acidurias.

## Materials and Methods

### Chemicals

Dulbecco’s modified Eagle’s medium with nutrient mixture F-12 with glucose content 17.5 mM (DMEM/F12; catalog number: D8437), bovine serum albumin (BSA), Dulbecco’s phosphate-buffered saline (DPBS), trypsine, sodium pyruvate, oligomycin, rotenone, carbonyl cyanide 3-chlorophenylhydrazone (CCCP), succinate, 3-(trimethylsilyl)propionic-2,2,3,3-d_4_ acid sodium salt, antimycine A, TRITON X-100 and other common chemical were purchased from Sigma-Aldrich (MO, USA), except to cytochrome c, and MiR05 those were from Oroboros AT (Innsbruck, Austria), and digitonin that was obtained from Fluka (Charlotte, US).

### Cell Culture

The cells of the human neuroblastoma cell line SH-SY5Y (ATCC-CRL-2266) were obtained from Sigma (MO, USA), and cultured according the supplier´s instructions. Briefly, the cells were cultured in mix of DMEM/F12 that was supplemented with 10% (v/v) fetal bovine serum (FBS), 50 U/ml penicillin and 50 mg/ml streptomycin. The cells were kept in incubator with humidified atmosphere with 5% CO_2_ at 37 °C. The culture media were renewed every 3 days before start of the experiment, and the cells were used for experiments after they reached confluency of at least 60% and up to a maximum 85%.

To incubate the cells in presence of either D-2HG or L-2HG, the media were refreshed and supplemented with appropriate enantiomer of 2HG at desired concentration. The incubations in presence of 2HG were carried out either for 24–48 h. In case of 48-h incubation, the media were refreshed after 24 h, to reset the level of 2HG. At the end of incubation, the collected media were clarified by centrifugation at 1500 x *g* for 10 min and subsequently stored at -20 °C. The adhered cells were rinsed twice with cold DPBS, and subsequently lysed with 50 mM Tris/HCl buffer with pH = 7.4, 1 mM EDTA, 1% (w/w) Triton X-100. The obtained cell lysates were stored at -20 °C before further analysis.

Three independent samples from group L-HG, and D-HG and four independent samples from control group were made on different days and within three cell passages. Firstly, the frozen stocks of cells in the same passage were reused to regrowth the cells to keep the limited number of passages. The cells were grown in plastic well-plates with different diameters to obtain sufficient amount of material for particular analysis.

### ^1^H-NMR Analysis

In 500 µL of clarified cell culture medium was mixed with 100 µL of deuterium oxide solution consisting of 200 mM phosphate buffer, pH 7.4 supplemented with 0.2 mM 3-(trimethylsilyl)propionic-2,2,3,3-d_4_ acid sodium salt. The mixture was transferred into NMR cuvette with diameter of 5 mm.

### ^1^H-NMR Data Acquisition and Processing

^1^H-NMR spectroscopy, the data acquisition and processing, were performed as already described (Baranovicova et al. 2018). To estimate the absolute concentrations of the identified metabolites the external standards were used.

### Enzymatic Assays

The enzymatic assays were performed to estimate the total and specific activities of lactate dehydrogenase (LDH) and 3-hydroxybutyrate dehydrogenase (3-HBDH), as well as to determine the concentration of 3-hydroxybutyrate (3-HB), and cell survival.

The reaction buffer (RB), used for the enzymatic assays consisted of 0.1 M glycine/NaOH buffer with pH 9 supplemented with 50 mM hydrazine and 5 mM NAD^+^. To assess the enzymatic activity of LDH or 3-HBDH, the RB was enriched up to the final concentration 10 mM either with lactate or 3-HB, respectively. For the assay, 30 µL of cell lysate or clarified medium was mixed with 250 µL of RB with addition of lactate or 3-HB, and the change of absorbance (λ = 340 nm) reflecting the formation of NADH in time was recorded and subsequently used to calculate the enzymatic activity of LDH and 3-HBDH.

The estimation of the concentration of 3-HB in culture media was performed with purified 3-HBDH as already described [17].

The cell survival was calculated as the ratio of LDH activity in cell lysates to total LDH activity according as already described [18].

### Protein Estimation

Protein content of cell lysates was estimated by Bradford assay with commercially supplied reagent, according the manufacturer’s instructions. Bovine serum albumin (Sigma-Aldrich MO, USA), was used as the protein standard.

### Determination of D-2HG and L-2HG Levels

The concentrations of the both enantiomers of 2HG in culture media were estimated as follows. The clarified culture media were derivatized [19] and subsequently applied on Phenomenex (Torrance, USA) Kinetex Polar C18 (3.0 mm x 150 mm; 2.6 μm particle size) column connected to Agilent 1260 HPLC equipped with quaternary pump module. For LC gradient eluent A was 2 mM ammonium formate, pH 3.60 and eluent B was acetonitrile (ACN). Gradient program was setup as 0–0.5 min-100% A, 0.5–8 min - linear gradient to 3.5% ACN, 8–16 min - linear gradient to 50% ACN, 3 min. 100% ACN column wash at flow rate 0.30 ml/min. The samples along with both L- and D-2HG standard sets (2 calibration sets, in a bracketing fashion) were injected into HPLC. The detection was done on Bruker Impact II Q-TOF HRMS spectrometer in MS-MS mode using Bruker (Zaandam, Netherlands) HyStar acquisition software. MS-MS EIC chromatograms were extracted for 363.05 -> 147.03 and 366.06 -> 150.05 m/z (IS) transitions. Obtained MS and MS-MS data were processed in Bruker QuantAnalysis module. So, the obtained MS data for L- and D-2HG peaks in EIC chromatograms were set for 363.05 ± 0.01 m/z, integrated and plotted as calibration curves separately for the L- and D- isomers (EIC chromatograms set for 366.06 ± 0.01 m/z for IS). Also, from MS-MS data L- and D-2HG peaks in EIC chromatograms were set for 147.03 ± 0.01 m/z (EIC chromatograms set for 150.05 ± 0.01 m/z for IS) and processed similarly.

### High-Resolution Respirometry

For an analysis of mitochondrial function in intact cells high-resolution respirometry measurements were performed in a two-chamber system O2k-FluoRespirometer (Oroboros AT, Innsbruck, Austria) according the already described method [20]. For the respiratory measurements, SH-SY5Y cells were incubated in presence of either of two 2HG enantiomers in the culture medium in level of 0.1 mM for 24 h. Cells detached by trypsinization were resuspended in respiration medium MiR05-Kit to cellular density of 10^6^ cells per one ml.

### The Satistical Analysis

The obtained data were statistically analyzed by One-way ANOVA statistic with Dunnett´s multiple comparison test and the results were considered statistically significantly different if *p* < 0.05.

## Results

**Fig. 1:**
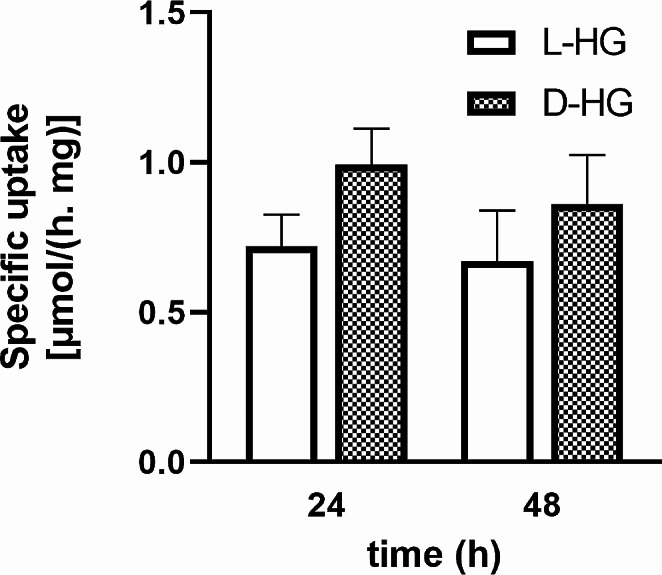
Human neuroblastoma cells (SH-SY5Y) were divided into two groups. The cells of the first and second groups were incubated with addition of L-HG or D-HG (0.1 mM) for 24 h (24) in culture medium. Subsequently, the culture media were replaced in both cases with fresh media enriched with 0.1 mM L-HG or D-HG (24 + 24). We quantified the specific uptake of both 2HG enantiomers from the culture medium by high-performance liquid chromatography (HPLC) with high-resolution mass spectrometry (HRMS). All values represent mean ± SD from three independent experiments

Several published observations reveal that D-2HG already in 0.1 mM level possesses the potential to affect several types of animal cells [21–24]. With the aim to evaluate the capability of the human neuroblastoma cells, SH-SY5Y, to dispose the both enantiomers, D or L, of 2HG from culture media, the cells were incubated in culture medium supplemented with either of the 2HG enantiomers at the initial level of 0.1 mM for 24–48 h. In case of the 48-hour incubation was applied, the media with L- or D-2HG were once refreshed in the middle time of the incubation. The remaining concentrations of both, D-2HG and L-2HG, in the culture media were estimated by HPLC-MS method. The neuroblastoma cells disposed D-2HG and L-2HG from culture media with specific rates 0.67 ± 0.24 and 0.86 ± 0.28 µmol·h^− 1^·mg^− 1^ of cellular proteins, respectively, which have not changed statistically significantly after prolonged incubation for 48 h (Fig. 1).

The supplementation of the culture media to 100 µM level of either L-2HG or D-2HG had no effect on the total amount of proteins and also on the survival of the cultured cells if estimated as the ratio of lactate dehydrogenase activity present in incubation media released from the dying cells to sum of the lactate dehydrogenase activities present in media and lysates (Table 1).

**Table 1:** Effect of 2-hydroxyglutarate on cell survival, total lysate protein content, and specific activity of both lactate (a_s_ LDH) and 3-hydroxybutyrate (a_s_ 3-HBDH) dehydrogenase. Human neuroblastoma cells (SH-SY5Y) were divided into four groups. The cells of the first and second groups were incubated for 24–48 h in medium supplemented with 0.1 mM L-2HG or D-2HG. Control group represents the cells cultivated in culture media without addition of L- or D-2HG. All values represent mean ± SD from at least three independent experiments (N) and * represents the value of *p* ≤ 0.05

Parameter (unit)	Control	L-2HG 100 µM		D-2HG 100 µM	
	24 h	24 h	48 h	24 h	48 h
**Cell survival (%)**	80 ± 5	84 ± 1	81 ± 6	82 ± 2	82 ± 1
**Protein content (mg)**	0.47 ± 0.07	0.541 ± 0.002	0.540 ± 0.004	0.54 ±0.07	0.53 ± 0.03
**a**_**s**_ **[LDH] (U/g)**	72 ± 13	64 ± 6	64 ± 12	55 ± 5*	61 ± 3
**a**_**s**_ **[3-HBDH] (U/g)**	0.88 ± 0.20	0.72 ± 0.06	1.57 ± 0.28*	1.10 ± 0.40	0.99 ± 0.11
**N**	4	3	3	3	3

The estimation of the specific lactate dehydrogenase activities in the lysates of the cells incubated in media supplemented with L-2HG or D-2HG enantiomers revealed their dual effect. While presence of D-2HG for 24 h inhibited specific lactate dehydrogenase activity in cells, the incubation of cells with D-2HG for 48 h or in presence of L-2HG for either 24–48 h had no inhibitory effect on specific activity of lactate dehydrogenase (Table 1).

**Fig. 2:**
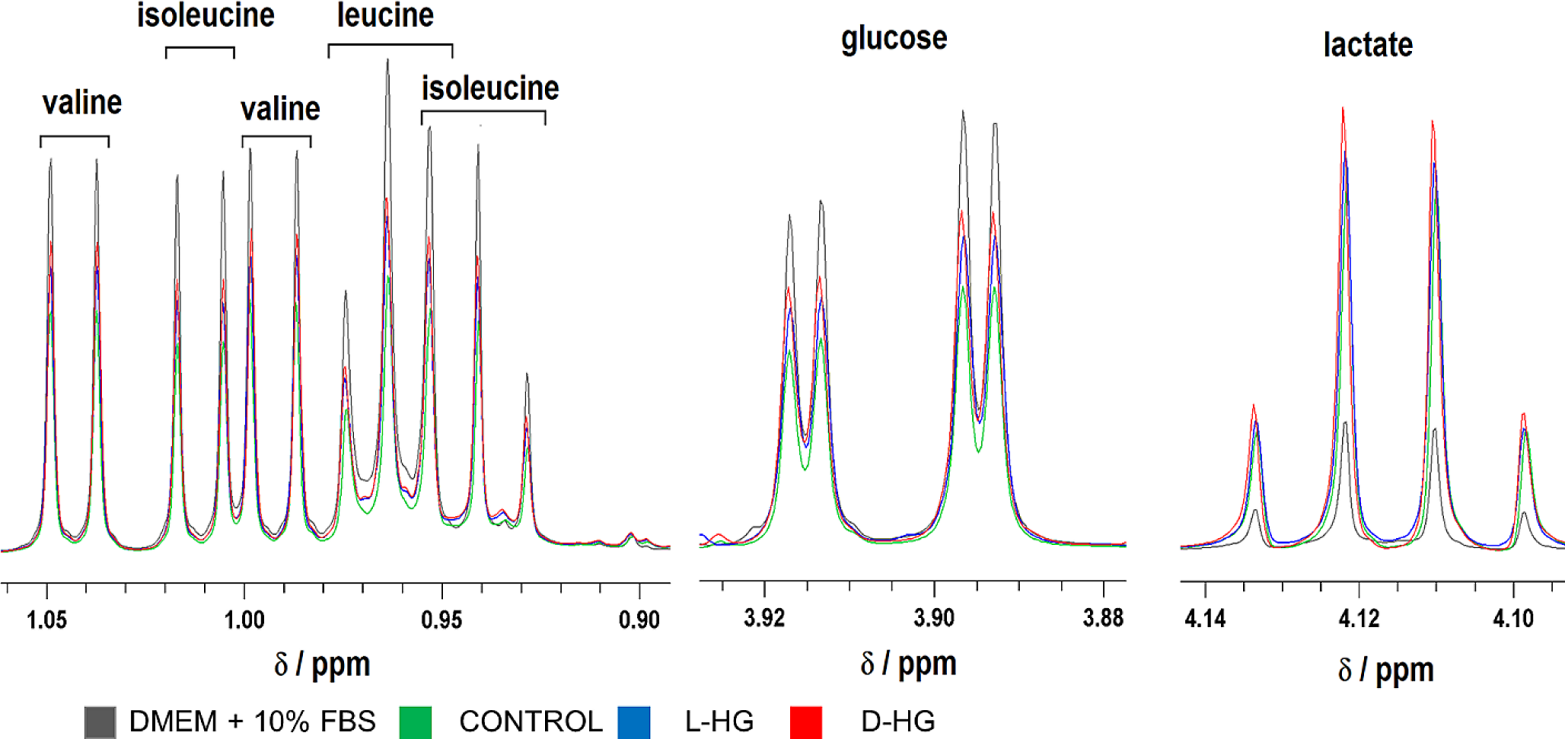
Representative spectra of glucose, lactate, and branched-chain amino acids, namely leucine, isoleucine, valine of culture media consisting of DMEM/F12 supplemented with 10% FBS without L or D-hydroxyglutarate (CONTROL, green), or with addition of 0.1 mM L-hydroxyglutarate (L-HG, blue), or with 0.1 mM D-hydroxyglutarate (D-HG, red) after 24 h incubation with neuroblastoma cells. Representative spectra of mentioned metabolites of culture media consisting of DMEM/F12 supplemented with 10% FBS without L or D-hydroxyglutarate before 24 h incubation (DMEM/F12 + 10% FBS, black) served as internal standard. All spectra were obtained by ^1^H-NMR spectroscopy and the ranges of chemical shifts (δ) for glucose, lactate, and all three BCAAs are depicted

The culture media were collected after 24-hour incubation, and subjected to ^1^H-NMR spectroscopy. The obtained spectra (Fig. 2, Fig. S1, S2) were used to quantify the remaining levels of the compounds composing the media, as well as the compounds released into the media. The obtained data were used to estimate the cellular metabolic capacity by their standardization to the total amount of cellular protein and time of the incubation (Table 2). The metabolomic analysis revealed that human neuroblastoma cells readily dispose the majority of the essential amino acids from culture medium, except lysine and tryptophan those levels remained almost unchanged (data not shown). Among essential amino acids, leucine, isoleucine, valine and threonine were disposed from culture media most potently, with the specific uptake rates 24 ± 5, 22 ± 5, 17 ± 5 and 17 ± 5 nmol*h^− 1^*mg^− 1^ of protein, respectively (Table 2).

**Table 2:** The measurement of specific uptake or released of metabolites from culture media derived from monitored groups by 1 H-NMR analysis. Human neuroblastoma cells (SH-SY5Y) were divided into four groups. The cells of the first and second groups were incubated for 24 h in medium with 0.1 mM L-2HG or D-2HG (24 h). In case of the 48-hour incubation, the media were once refreshed with the medium with L- or D-2HG in the middle time of the incubation (48 h). Control group represents the cells cultivated in culture media without addition of L- or D-2HG. Specific uptake or release (negative value) of metabolite from culture media was calculated by their standardization to the total amount of cellular protein and time of the incubation (nmol*h− 1*mg− 1). All values represent mean ± SD from at least three independent experiments (N) and * represents the value of *p* ≤ 0.05

Metabolite	Control	L-2HG 100 µM		D-2HG 100 µM	
	24 h	24 h	48 h	24 h	48 h
**N**	4	3	3	3	3
**glucose**	-518 ± 141	-302 ± 22	-389 ± 113	-223 ± 66	-332 ± 52
**lactate**	135 ± 23	158 ± 9	147 ± 11	142 ± 14	135 ± 7
**pyruvate**	-26 ± 5	-21 ± 3	-23 ± 2	-21 ± 5	-26 ± 2
**alanine**	7 ± 2	13 ± 1	16 ± 2	15 ± 1	13 ± 1
**acetate**	3 ± 2	3 ± 1	3.1 ± 0.2	2.8 ± 0.4	3.3 ± 0.5
**formate**	336 ± 45	378 ± 24	341 ± 22	369 ± 29	350 ± 23
**leucine**	-24 ± 5	-16.2 ± 0.4	-18 ± 4	-14 ± 3	-18 ± 2
**isoleucine**	-22 ± 5	-15.2 ± 0.3	-16 ± 4	-12 ± 2	-16 ± 2
**valine**	-17 ± 5	-9 ± 1	-11 ± 4	-7 ± 2	-11 ± 1
**glutamine**	-131 ± 38	-94 ± 1	-91 ± 41	-86 ± 18	-109 ± 9
**pyroglutamate**	-12 ± 8	2 ± 2	-5 ± 2	8 ± 3	2 ± 6
**threonine**	-17 ± 10	-7 ± 3	-7 ± 3	-2 ± 5	-6 ± 5
**phenylalanine**	-7 ± 3	-2.1 ± 0.1	-4 ± 3	-1 ± 1	-3 ± 1
**methionine**	-4 ± 2	-1.4 ± 0.2	-2 ± 1	-1 ± 1	-2.4 ± 0.2
**histidine**	-8 ± 2	-3 ± 1	-6 ± 4	-3 ± 1	-5 ± 1
**tyrosine**	-6 ± 3	-2.2 ± 0.3	-3 ± 2	-1 ± 1	-3 ± 1

**Fig. 3:**
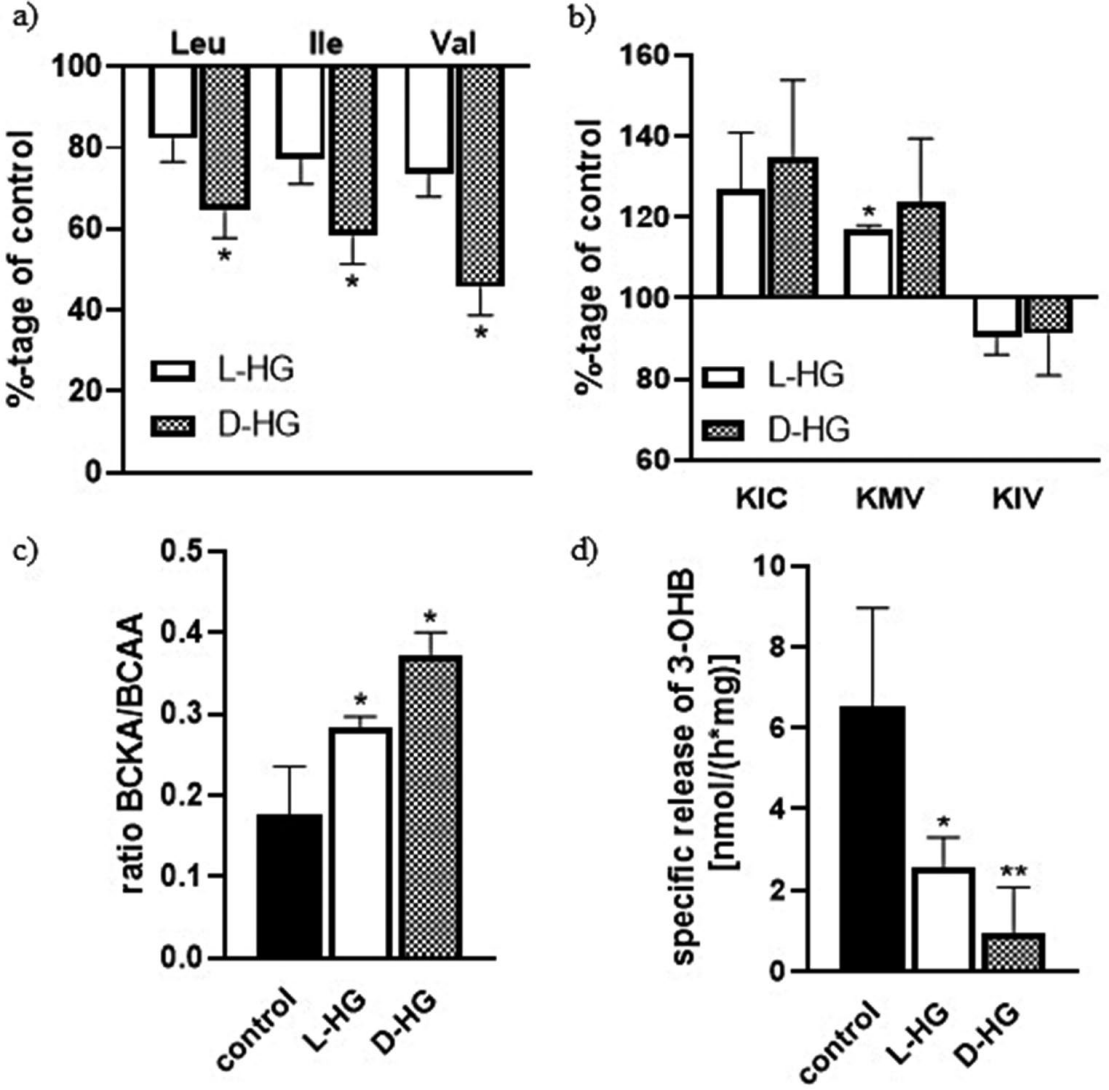
The effect of 2-HG enantiomers on removal of branched-chain amino acids from culture media (BCAA; a), enrichment of media with branched-chain keto acids (BCKA; b) and 3-hydroxybutyrate (3-OHB; d), as well as the ratio BCKA/BCAA (c). Human neuroblastoma cells (SH-SY5Y) were subdivided into three groups. The cells of the first and second groups were incubated for 24 h in medium with L-HG or D-HG (0.1 mM). The third group, the control, was incubated under the same conditions but without the addition of 2HG. These media were subjected to metabolomic analysis by ^1^H-NMR after 24 h of incubation. The control group, in figure a, and b represents 100%. The abbreviations used are: KIC, α-ketoisocaproate; KMV, α-keto-methylvalerate; KIV, α-ketoisovalerate. All values represent mean ± SD from at least three independent experiments

**Fig. 4:**
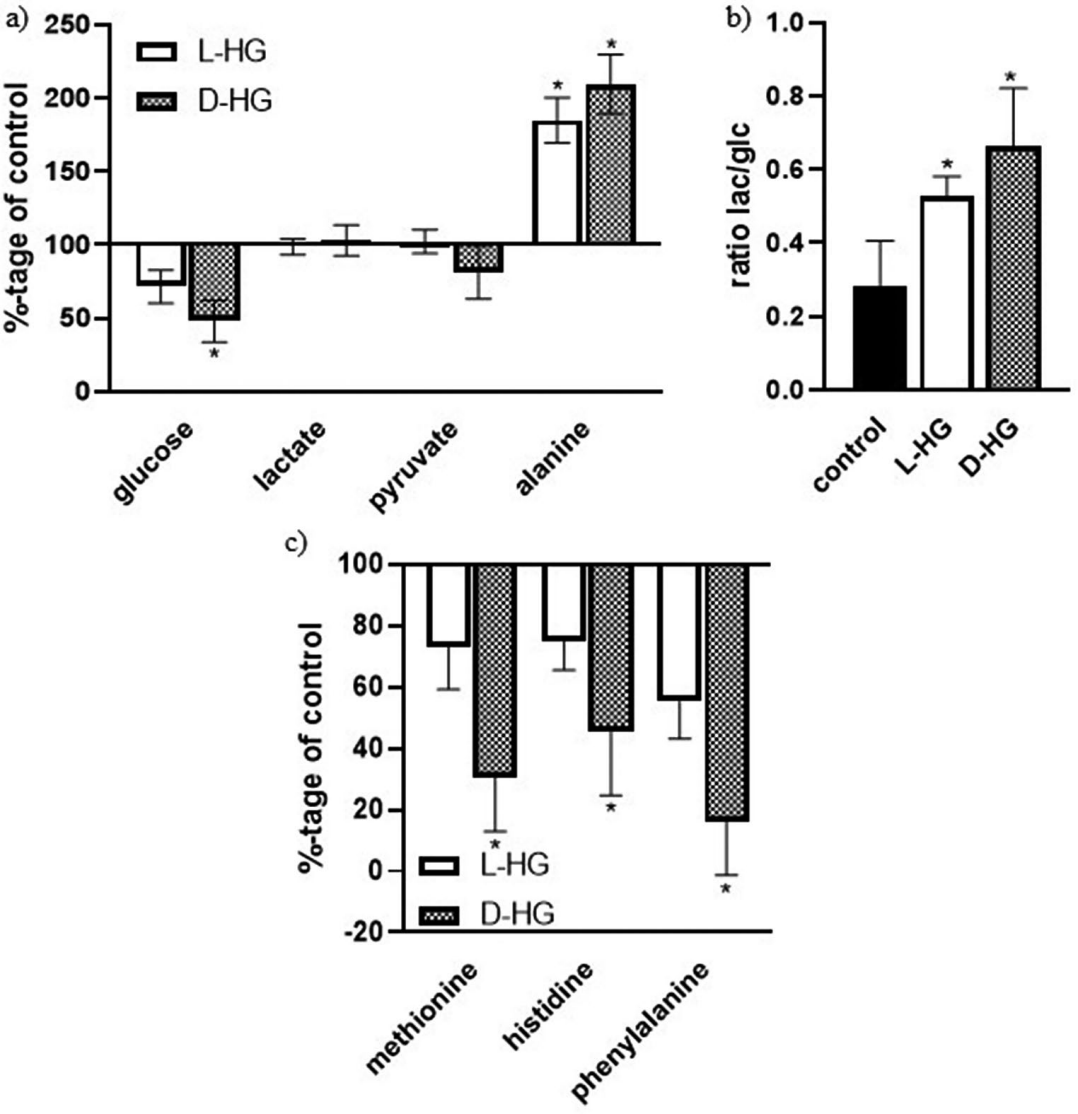
The effect of 2-HG enantiomers on levels of glucose (glc), lactate (lac), pyruvate and alanine (a) and three essential amino acids – methionine, histidine and phenylalanine (c) in culture media and the estimated ratio of lac/glc (b). Human neuroblastoma cells (SH-SY5Y) were divided into three groups. The cells of the first and second groups were incubated for 24 h in medium with L-HG or D-HG (0.1 mM). The third group, the control, was incubated under the same conditions but without the addition of 2HG. These culture media were subjected to metabolomic analysis by ^1^H-NMR after 24 h of incubation. The third group, the control group, represents 100% (a, and c). All values represent mean ± SD from at least three independent experiments

The presence of the both enantiomers of 2HG in culture media either for 24–48 h, affected the cellular capacity to dispose some of the essential amino acids from culture media to different extent. The measured values show that the enantiomers of 2HG slowed down the metabolism of branched-chain essential amino acids (BCAA, Fig. 3a), while the amount of their respective keto acids remained the same or the responsible keto acid for isoleucine, 2-oxo-methylvalerate, was significantly increased by influence of L-2HG (Fig. 3b). The calculated ratio of the amount of BCKA released into the culture medium and the amount of metabolized BCAAs from the culture medium, was significantly increased by the effect of both L- and D-2HG (Fig. 3c). From these results we deduce a slowing down of BCKA metabolism down to the end metabolites of their metabolism, which was confirmed by a significant reduction in the release of 3-hydroxybutyrate into the culture medium under the influence of L and D 2HG (Fig. 3d). In addition to the slowed metabolism of BCAA, we also observed a decreased metabolism of other essential amino acids (methionine, phenylalanine, histidine) in the culture medium due to effect of both enantiomers of 2HG (Fig. 4c).

At the same time, we observed reduced glucose uptake from the culture medium, while the amount of metabolites released into the culture medium from glucose metabolism (lactate, pyruvate) remained unchanged under the influence of L and D 2HG (Fig. 4a). Calculating the ratio of the amount of lactate released to the amount of glucose taken up from the culture medium, we found that both enantiomers of 2HG increase anaerobic glycolysis in a neuroblastoma cell line (Fig. 4b). Also influence of both enantiomers increase level of alanine in culture media, which means less free pyruvate for entrance to TCA cycle (Fig. 4a).

**Fig. 5:**
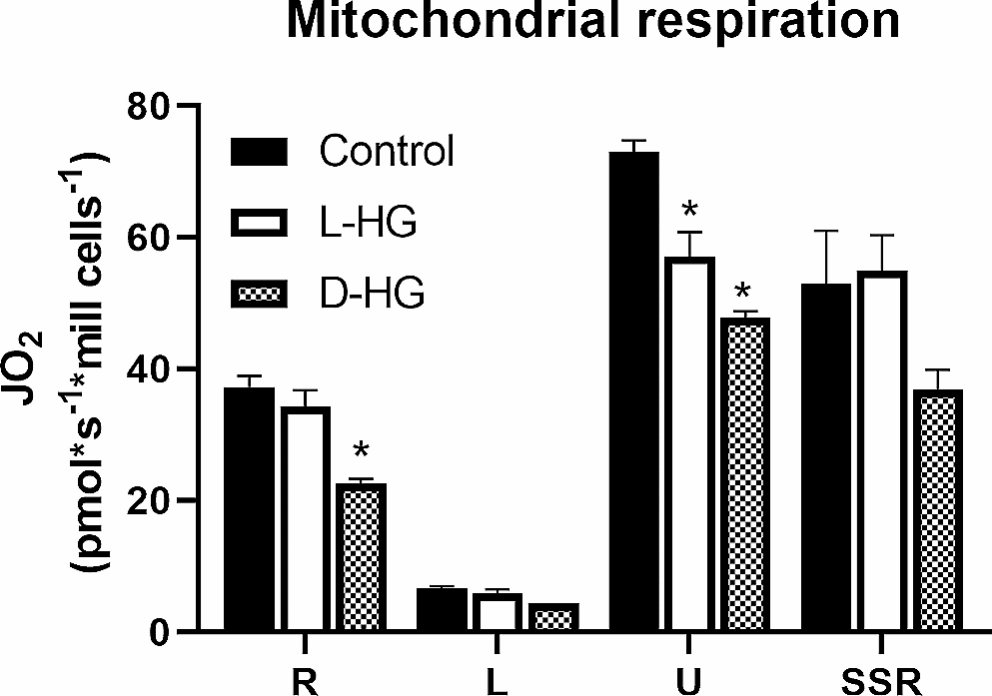
Impact of L-HG or D-HG on mitochondrial respiration parameters of human neuroblastoma cells (SH-SY5Y). Human neuroblastoma cells (SH-SY5Y) were divided into three groups. The cells of the first and second groups were incubated for 24 h in medium with L-HG or D-HG (0.1 mM). The third group, the control, was incubated under the same conditions but without the addition of 2HG.The graphical representation compares mitochondrial respiration among three groups, and resulting O2 flux values were employed to calculate the following parameters: basal respiration (R), leak (L), maximal electron transport capacity (U), succinate stimulated respiration (SSR). Displayed results are corrected to residual oxygen consumption (ROX). All values represent mean ± SD from three independent experiments and *represents the value of *p* ≤ 0.05

The respiration capacity of the cells was estimated by measuring the oxygen consumption. The specific basal rate of respiration was 37 ± 2 pmol*s^− 1^ per 10^6^ cells. The incubation of the cells in presence of D-2HG at the level of 0.1 mM in culture media for 24 h suppressed the specific basal respiration capacity of the cells for 40%. The second optical isomer, L-enantiomer of 2HG, had not an effect on specific basal respiration. The both enantiomers, D and L, decreased the maximal specific respiration capacity of the cells to 66% or 78% of the untreated, control cells. Maximal specific respiration capacity of the control cells was 73 ± 3 pmol*s^− 1^ per 10^6^ cells (Fig. 5).

## Discussion

The broad range of neurological symptoms and even progressive damage of neural tissue are associated with elevated levels of 2HG in the brain parenchyma [3–5, 8, 14]. The studies on different animal models revealed several cellular and molecular targets for 2HG. The observed cytotoxic effect of either of 2HG enantiomers on animal neural cells or brains was associated with the capability of 2HG to inhibit 2-oxoglutarate-dependent enzymes, stimulate oxidative stress, inhibit electron transfer in the respiratory chain and impair energy metabolism, alter redox status of cells and several signaling cascades.

Previous experiments focusing on the cytotoxic effect of 2HG showed that a 0.1 mM level of D-2HG supplementation in culture media for a day possesses the potential to initiate the death of a significant proportion of animal neuronal cells [21–24]. The cytotoxic effect of L-2HG on animal neuronal cells was obtained by supplementing the media to at least 10 mM level [24]. The supplementation of culture media with either of two isoforms of 2HG at the level 0.1 mM had no effect on the cell survival rate or total protein content, even after incubation for 48 h. In patients suffering from 2-hydroxyglutaric acidurias, the levels of D-2HG or L-2HG in cerebrospinal fluid could reach up to 0.2 mM or 0.5 mM, respectively [3]. In our case, the absence of the cytotoxic effect of 2HG on human neuroblastoma cells could reflect the interspecies difference in 2HG toxicity. Indeed, the controversies about the cytotoxic effect of 2HG on human brain cells could be drawn based on the clinical observations. While it was concluded that the association between an increased level of 2HG in cerebrospinal fluid and the severity of brain atrophy pointed to the neurotoxic effect of 2HG, the observation that brain atrophy is observable also in patient suffering 2HG aciduria even with the physiological level of 2HG in cerebrospinal fluid [3, 4, 7].

Both optical isomers of 2HG are considered to influence the cellular metabolism of cultured cells. We analyzed the effect of either of 2HG isomers on the metabolism of human neuroblastoma cells by analysis of their media composition by ^1^H-NMR. The both isomers of 2HG if present in media for 24 h affect the metabolism of human cells. The quantification of glucose uptake from culture media and subsequent release of lactate, pyruvate and alanine from SH-SY5Y cells revealed the potential of 2HG to decrease the glucose consumption without affecting the cellular capability to release lactate. In this way, the estimated molar ratios of released lactate to consumed glucose molecules were increased, representing the increased rate of anaerobic metabolism. Indeed, the potential of 2HG to inhibit mitochondrial respiration in several types of animal cells and in cells of human embryonic kidney cell line HEK 293 has been already documented. The cellular respiration was affected by capability of 2HG to block some of the complexes in the respiratory chain [10, 24]. In our experimental set up, the preincubation of neuroblastoma cells in media with 2HG lead to suppression of the total respiration capacity of cells, without affecting the activities of particular complexes. In addition, the effect of unphysiologically high glucose concentration in culture medium were not further elucidated.

Besides glucose, amino acids are considered as to serve a role of the complementary substrates for cellular metabolism of the cells in neuronal tissue. Out of essential proteinogenic amino acids, leucine, isoleucine and valine are considered to be imported with appropriately high rate into the brain parenchyma from blood stream through blood-brain barrier and subsequently withdrawn into the brain cells from their milieu. Indeed, all three amino acids were withdrawn by cultured neuroblastoma cells with the rates exceeding all other amino acids [17]. The catabolism of BCAA provides the cells with substantial amount of nitrogen and carbon atoms. While nitrogen atoms in form of amino group could be used for synthesis of amino acids, especially in brain parenchyma, BCAA are considered the significant source of nitrogen atoms present in structures of de novo synthesized molecules of glutamate and glutamine. The carbon skeleton of leucine can enter the catabolism in brain cells and may be catabolized to acetoacetate that is further reduced to 3-hydroxybutyrate. The both last molecules are ketone bodies. Indeed, the production of ketone bodies by human brain cancer cells correlates with catabolic flux of leucine. In presence of 2HG the capacity of neuroblastoma cells to sink the level of BCAA in culture media is substantially decreased and together with pronounced rise of released amount of their cognate 2-oxo acids into media correlate with suppressed formation of 3-hydroxybutyrate. While these findings suggest a potential inhibition of the enzymatic conversion of BCAA-derived carbon skeletons in the irreversible part of their catabolism, it’s also possible that 2-HG could impact the activity and/or expression of corresponding transporters.

Impaired metabolism of BCAA could affect the rate of neurotransmitter synthesis or recycling, such as glutamate and its glutamate – glutamine cycle. The disbalance of glutamate metabolism and possibly also its neurotransmitter role might contribute to development of neurological signs associated with 2HG disbalance, such as epilepsy or ataxia [25]. In this respect, it remains to be estimated, to which extend may 2HG affect the generation of ketone bodies in brain parenchyma and furthermore, what could be a consequence of the lower production of 3-hydroxybutyrate in etiopathogenesis of neurological and neurodegenerative symptoms associated with 2HG acidurias. Even though the details about metabolic, signaling and regulatory roles of 3-hydroxybutyrate in human brain remains uncovered [26] they can be sources of acetyl moieties for sustaining the energy metabolism and lipid synthesis in oligodendrocytes, as well as signaling molecules for suppressing the epileptic episodes. Furthermore, ketone bodies could contribute to the synthesis of cytosolic acetyl-CoA molecules, which are also needed for acetylation reactions in the cytosol, including the nucleus.

Brain cells are known to produce small amounts of 2HG enantiomers [6]. Also, human neuroblastoma cells SH-SY5Y has been shown to generate both optical isomers of 2HG in culture, which production can be affected by inhibition of cellular respiration [6, 27, 28]. Brain cell are equipped with 2HG dehydrogenases those allowed for oxidation of 2HG to 2-oxoglutarate [29, 30]. Disturbances in the activity of either of the isoforms of 2HG dehydrogenase initiate a rise in level of corresponding 2HG isomer due to its insufficient degradation [3, 8, 14]. Several transporters are capable of transporting 2HG molecules through membranes [31], which facilitate the translocation of ionic molecules of 2HG in both directions. The cell-specific distribution of 2HG transporters among neural cells is not well established. However, the expression of 2HG transporters by SH-SY5Y cells could be deduced based on their capability to remove both enantiomers of 2HG from their culture media.

The estimated specific uptake rate for both stereoisomers of 2HG seems to be insufficient to compensate the loss of the carbon and nitrogen atoms in metabolism of neuroblastoma cells due to lowered uptake of glucose and amino acids together with increased release of lactate and alanine. In this respect, it could be hypothesized that the metabolism of neuroblastoma cells may also engage on other source of carbon and nitrogen atoms – extracellular proteins. Such deduction agrees with the published data, which clearly identify the capacity of several types of cancer cells to enhance their macropinocytotic potential for extracellular protein to enhance the uptake of substrates for their metabolism. However, it remains to be established to which extend can human neuroblastoma cells use extracellular protein as the source of substrates for their metabolism and to what degree could be such process affected by 2HG (unpublished data).

The neuroblastoma cells SH-SY5Y are commonly applied model to study the cytotoxic effects of compounds on human neurons and molecular mechanisms underlying the neurodegeneration [32–35]. The limitation in use of neuroblastoma cells as the study model is their cancerous origin. Therefore, in vitro results obtained with a such study model provides the basis for further confirmation on more complex cellular, animal models or even by well-considered intervention in patients.

## Conclusion

The use of human neuroblastoma cells SH-SY5Y to evaluate the effect of 2HG on metabolism of human neural cells appears to represent the appropriate study model. Even the survival rate of tested cells remained unaffected, the both enantiomers of 2-HG affected several metabolic features mostly associated with promoting the anaerobic metabolism, impacting some of the cellular respiration aspects, and capability to metabolize several of amino acids. Such biochemical and metabolic alterations could underlie the changes on molecular and cellular levels leading to pathological manifestation associated with increased 2-HG levels.

### Electronic Supplementary Material

Below is the link to the electronic supplementary material.

Supplementary Material 1

## Data Availability

No datasets were generated or analysed during the current study.
