# Supplementary material for: Both Enantiomers of 2-Hydroxyglutarate Modulate the Metabolism of Cultured Human Neuroblastoma Cells
Source: Neurochem Res. 2024 Jun 12;49(9):2480–90. doi: 10.1007/s11064-024-04188-8 (PMC11310259; doi:10.1007/s11064-024-04188-8)
Supplement: Supplementary file 1 — Supplementary Material 1 [file 11064_2024_4188_MOESM1_ESM.docx]

**Table S1a-h.** The analysis of variance tables for data analysis presented in **Table 1**. The presented are values of the sum of squares due to source (SS), the degree of freedom in the source (DF), the mean sum of squares due to the source (MS), the F-statistic with values for degrees of freedom numerator (DFn) and degrees of freedom denominator (DFd) in brackets. The table is subdivided into parts **a – h** based on the source of the variation in the data (**Source**).

| 1. **Source: Table 1** | **Cell survival** | **24h** |  |  |  |
| --- | --- | --- | --- | --- | --- |
| ANOVA table | SS | DF | MS | F (DFn, DFd) | P value |
| Treatment (between columns) | 10,83 | 2 | 5,417 | F (2, 7) = 0,4136 | P=0,6764 |
| Residual (within columns) | 91,67 | 7 | 13,1 |  |  |
| Total | 102,5 | 9 |  |  |  |
|  |  |  |  |  |  |
| 1. **Source: Table 1** | **Cell survival** | **48h** |  |  |  |
| ANOVA table | SS | DF | MS | F (DFn, DFd) | P value |
| Treatment (between columns) | 2,733 | 2 | 1,367 | F (2, 7) = 0,05706 | P=0,9450 |
| Residual (within columns) | 167,7 | 7 | 23,95 |  |  |
| Total | 170,4 | 9 |  |  |  |
|  |  |  |  |  |  |
| 1. **Source: Table 1** | **protein** | **24h** |  |  |  |
| ANOVA table | SS | DF | MS | F (DFn, DFd) | P value |
| Treatment (between columns) | 0,02214 | 2 | 0,011 | F (2, 7) = 3,925 | P=0,0719 |
| Residual (within columns) | 0,01974 | 7 | 0,003 |  |  |
| Total | 0,04189 | 9 |  |  |  |
|  |  |  |  |  |  |
| 1. **Source: Table 1** | **protein** | **48h** |  |  |  |
| ANOVA table | SS | DF | MS | F (DFn, DFd) | P value |
| Treatment (between columns) | 0,01753 | 2 | 0,009 | F (2, 7) = 4,530 | P=0,0547 |
| Residual (within columns) | 0,01355 | 7 | 0,002 |  |  |
| Total | 0,03108 | 9 |  |  |  |
|  |  |  |  |  |  |
| 1. **Source: Table 1** | **a_s_ [LDH]** | **24h** |  |  |  |
| ANOVA table | SS | DF | MS | F (DFn, DFd) | P value |
| Treatment (between columns) | 318,2 | 2 | 159,1 | F (2, 7) = 4,442 | P=0,0568 |
| Residual (within columns) | 250,7 | 7 | 35,81 |  |  |
| Total | 568,9 | 9 |  |  |  |
|  |  |  |  |  |  |
| 1. **Source: Table 1** | **a_s_ [LDH]** | **48h** |  |  |  |
| ANOVA table | SS | DF | MS | F (DFn, DFd) | P value |
| Treatment (between columns) | 120,3 | 2 | 60,17 | F (2, 7) = 0,9814 | P=0,4210 |
| Residual (within columns) | 429,2 | 7 | 61,32 |  |  |
| Total | 549,6 | 9 |  |  |  |
|  |  |  |  |  |  |
| 1. **Source: Table 1** | **a_s_ [3-HBDH]** | **24h** |  |  |  |
| ANOVA table | SS | DF | MS | F (DFn, DFd) | P value |
| Treatment (between columns) | 0,227 | 2 | 0,114 | F (2, 7) = 1,844 | P=0,2273 |
| Residual (within columns) | 0,4308 | 7 | 0,062 |  |  |
| Total | 0,6578 | 9 |  |  |  |
|  |  |  |  |  |  |
| 1. **Source: Table 1** | **a_s_ [3-HBDH]** | **48h** |  |  |  |
| ANOVA table | SS | DF | MS | F (DFn, DFd) | P value |
| Treatment (between columns) | 0,72 | 2 | 0,36 | F (2, 7) = 9,183 | P=0,0110 |
| Residual (within columns) | 0,2744 | 7 | 0,039 |  |  |
| Total | 0,9944 | 9 |  |  |  |

**Table S2.** The analysis of variance table for data analysis presented on **Figure 1**. The presented are values of the sum of squares due to source (SS), the degree of freedom in the source (DF), the mean sum of squares due to the source (MS), the F-statistic with in brackets values for degrees of freedom numerator (DFn) and degrees of freedom denominator (DFd).

| **Source: Figure 1** |  |  |  |  |  |
| --- | --- | --- | --- | --- | --- |
| ANOVA table | SS (Type III) | DF | MS | F (DFn, DFd) | P value |
| Interaction | 0,004326 | 1 | 0,004326 | F (1, 7) = 0,08114 | P=0,7840 |
| Row Factor | 0,02173 | 1 | 0,02173 | F (1, 7) = 0,4076 | P=0,5435 |
| Column Factor | 0,1427 | 1 | 0,1427 | F (1, 7) = 2,678 | P=0,1458 |
| Residual | 0,3732 | 7 | 0,05331 |  |  |

**Table S3a-h.** The analysis of variance tables for data analysis presented on **Figure 3**. The presented are values of the sum of squares due to source (SS), the degree of freedom in the source (DF), the mean sum of squares due to the source (MS), the F-statistic with values for degrees of freedom numerator (DFn) and degrees of freedom denominator (DFd) in brackets. The table is subdivided into parts **a – h** based on the source of the variation in the data (**Source**).

| 1. **Source: Figure 3a** | **Leucine** |  |  |  |  |
| --- | --- | --- | --- | --- | --- |
| ANOVA table | SS | DF | MS | F (DFn, DFd) | P value |
| Treatment (between columns) | 172,2 | 2 | 86,11 | F (2, 7) = 5,581 | P=0,0355 |
| Residual (within columns) | 108 | 7 | 15,43 |  |  |
| Total | 280,2 | 9 |  |  |  |
|  |  |  |  |  |  |
| 1. **Source: Figure 3a** | **Isoleucine** |  |  |  |  |
| ANOVA table | SS | DF | MS | F (DFn, DFd) | P value |
| Treatment (between columns) | 192,6 | 2 | 96,28 | F (2, 7) = 7,650 | P=0,0173 |
| Residual (within columns) | 88,09 | 7 | 12,58 |  |  |
| Total | 280,6 | 9 |  |  |  |
|  |  |  |  |  |  |
| 1. **Source: Figure 3a** | **Valine** |  |  |  |  |
| ANOVA table | SS | DF | MS | F (DFn, DFd) | P value |
| Treatment (between columns) | 172,1 | 2 | 86,07 | F (2, 7) = 6,968 | P=0,0216 |
| Residual (within columns) | 86,46 | 7 | 12,35 |  |  |
| Total | 258,6 | 9 |  |  |  |
|  |  |  |  |  |  |
| 1. **Source: Figure 3b** | **KIC** |  |  |  |  |
| ANOVA table | SS | DF | MS | F (DFn, DFd) | P value |
| Treatment (between columns) | 2,136 | 2 | 1,068 | F (2, 7) = 3,485 | P=0,0891 |
| Residual (within columns) | 2,145 | 7 | 0,3065 |  |  |
| Total | 4,281 | 9 |  |  |  |
|  |  |  |  |  |  |
| 1. **Source: Figure 3b** | **KMV** |  |  |  |  |
| ANOVA table | SS | DF | MS | F (DFn, DFd) | P value |
| Treatment (between columns) | 1,304 | 2 | 0,6521 | F (2, 7) = 4,549 | P=0,0542 |
| Residual (within columns) | 1,003 | 7 | 0,1433 |  |  |
| Total | 2,307 | 9 |  |  |  |
|  |  |  |  |  |  |
| 1. **Source: Figure 3b** | **KIV** |  |  |  |  |
| ANOVA table | SS | DF | MS | F (DFn, DFd) | P value |
| Treatment (between columns) | 0,5986 | 2 | 0,2993 | F (2, 7) = 4,455 | P=0,0565 |
| Residual (within columns) | 0,4703 | 7 | 0,06718 |  |  |
| Total | 1,069 | 9 |  |  |  |

| 1. **Source: Figure 3c** | **BCKA/BCAA** |  |  |  |  |
| --- | --- | --- | --- | --- | --- |
| ANOVA table | SS | DF | MS | F (DFn, DFd) | P value |
| Treatment (between columns) | 0,06587 | 2 | 0,03294 | F (2, 7) = 18,85 | P=0,0015 |
| Residual (within columns) | 0,01223 | 7 | 0,001747 |  |  |
| Total | 0,0781 | 9 |  |  |  |
|  |  |  |  |  |  |
| 1. **Source: Figure 3d** | **3-OHB** |  |  |  |  |
| ANOVA table | SS | DF | MS | F (DFn, DFd) | P value |
| Treatment (between columns) | 45,78 | 2 | 22,89 | F (2, 7) = 12,77 | P=0,0046 |
| Residual (within columns) | 12,54 | 7 | 1,792 |  |  |
| Total | 58,32 | 9 |  |  |  |

**Table S4a-h.** The analysis of variance tables for data analysis presented on **Figure 4**. The presented are values of the sum of squares due to source (SS), the degree of freedom in the source (DF), the mean sum of squares due to the source (MS), the F-statistic with values for degrees of freedom numerator (DFn) and degrees of freedom denominator (DFd) in brackets. The table is subdivided into parts **a – h** based on the source of the variation in the data (**Source**).

| 1. **Source: Figure 4a** | **glucose** |  |  |  |  |
| --- | --- | --- | --- | --- | --- |
| ANOVA table | SS | DF | MS | F (DFn, DFd) | P value |
| Treatment (between columns) | 156650 | 2 | 78325 | F (2, 7) = 7,392 | P=0,0188 |
| Residual (within columns) | 74169 | 7 | 10596 |  |  |
| Total | 230819 | 9 |  |  |  |
|  |  |  |  |  |  |
| 1. **Source: Figure 4a** | **lactate** |  |  |  |  |
| ANOVA table | SS | DF | MS | F (DFn, DFd) | P value |
| Treatment (between columns) | 898,7 | 2 | 449,3 | F (2, 7) = 1,656 | P=0,2577 |
| Residual (within columns) | 1899 | 7 | 271,3 |  |  |
| Total | 2798 | 9 |  |  |  |
|  |  |  |  |  |  |
| 1. **Source: Figure 4a** | **pyruvate** |  |  |  |  |
| ANOVA table | SS | DF | MS | F (DFn, DFd) | P value |
| Treatment (between columns) | 44,93 | 2 | 22,46 | F (2, 7) = 1,369 | P=0,3149 |
| Residual (within columns) | 114,8 | 7 | 16,41 |  |  |
| Total | 159,8 | 9 |  |  |  |
|  |  |  |  |  |  |
| 1. **Source: Figure 4a** | **alanine** |  |  |  |  |
| ANOVA table | SS | DF | MS | F (DFn, DFd) | P value |
| Treatment (between columns) | 55,21 | 2 | 27,6 | F (2, 7) = 12,78 | P=0,0046 |
| Residual (within columns) | 15,12 | 7 | 2,16 |  |  |
| Total | 70,33 | 9 |  |  |  |
|  |  |  |  |  |  |
| 1. **Source: Figure 4a** | **lac/glc** |  |  |  |  |
| ANOVA table | SS | DF | MS | F (DFn, DFd) | P value |
| Treatment (between columns) | 0,2616 | 2 | 0,131 | F (2, 7) = 9,299 | P=0,0107 |
| Residual (within columns) | 0,09845 | 7 | 0,014 |  |  |
| Total | 0,36 | 9 |  |  |  |
|  |  |  |  |  |  |
| 1. **Source: Figure 4a** | **methionine** |  |  |  |  |
| ANOVA table | SS | DF | MS | F (DFn, DFd) | P value |
| Treatment (between columns) | 13,13 | 2 | 6,564 | F (2, 7) = 4,356 | P=0,0590 |
| Residual (within columns) | 10,55 | 7 | 1,507 |  |  |
| Total | 23,68 | 9 |  |  |  |
|  |  |  |  |  |  |
| 1. **Source: Figure 4a** | **histidine** |  |  |  |  |
| ANOVA table | SS | DF | MS | F (DFn, DFd) | P value |
| Treatment (between columns) | 46,24 | 2 | 23,12 | F (2, 7) = 7,057 | P=0,0210 |
| Residual (within columns) | 22,93 | 7 | 3,276 |  |  |
| Total | 69,17 | 9 |  |  |  |
|  |  |  |  |  |  |
| 1. **Source: Figure 4a** | **phenylalanine** |  |  |  |  |
| ANOVA table | SS | DF | MS | F (DFn, DFd) | P value |
| Treatment (between columns) | 60,63 | 2 | 30,32 | F (2, 7) = 7,979 | P=0,0157 |
| Residual (within columns) | 26,6 | 7 | 3,799 |  |  |
| Total | 87,23 | 9 |  |  |  |

**Table S5a-d.** The analysis of variance tables for data analysis presented on **Figure 5**. The presented are values of the sum of squares due to source (SS), the degree of freedom in the source (DF), the mean sum of squares due to the source (MS), the F-statistic with values for degrees of freedom numerator (DFn) and degrees of freedom denominator (DFd) in brackets. The table is subdivided into parts **a – h** based on the source of the variation in the data (**Source**).

| 1. **Source: Figure 5** | R |  |  |  |  |
| --- | --- | --- | --- | --- | --- |
| ANOVA table | SS | DF | MS | F (DFn, DFd) | P value |
| Treatment (between columns) | 266,7 | 2 | 133,4 | F (2, 6) = 12,57 | P=0,0072 |
| Residual (within columns) | 63,66 | 6 | 10,61 |  |  |
| Total | 330,4 | 8 |  |  |  |
|  |  |  |  |  |  |
| 1. **Source: Figure 5** | L |  |  |  |  |
| ANOVA table | SS | DF | MS | F (DFn, DFd) | P value |
| Treatment (between columns) | 2,67 | 2 | 1,335 | F (2, 6) = 1,295 | P=0,3408 |
| Residual (within columns) | 6,185 | 6 | 1,031 |  |  |
| Total | 8,854 | 8 |  |  |  |
|  |  |  |  |  |  |
| 1. **Source: Figure 5** | U |  |  |  |  |
| ANOVA table | SS | DF | MS | F (DFn, DFd) | P value |
| Treatment (between columns) | 796,4 | 2 | 398,2 | F (2, 6) = 22,27 | P=0,0017 |
| Residual (within columns) | 107,3 | 6 | 17,88 |  |  |
| Total | 903,6 | 8 |  |  |  |
|  |  |  |  |  |  |
| 1. **Source: Figure 5** | SSR |  |  |  |  |
| ANOVA table | SS | DF | MS | F (DFn, DFd) | P value |
| Treatment (between columns) | 405,8 | 2 | 202,9 | F (2, 6) = 3,312 | P=0,1074 |
| Residual (within columns) | 367,6 | 6 | 61,27 |  |  |
| Total | 773,4 | 8 |  |  |  |


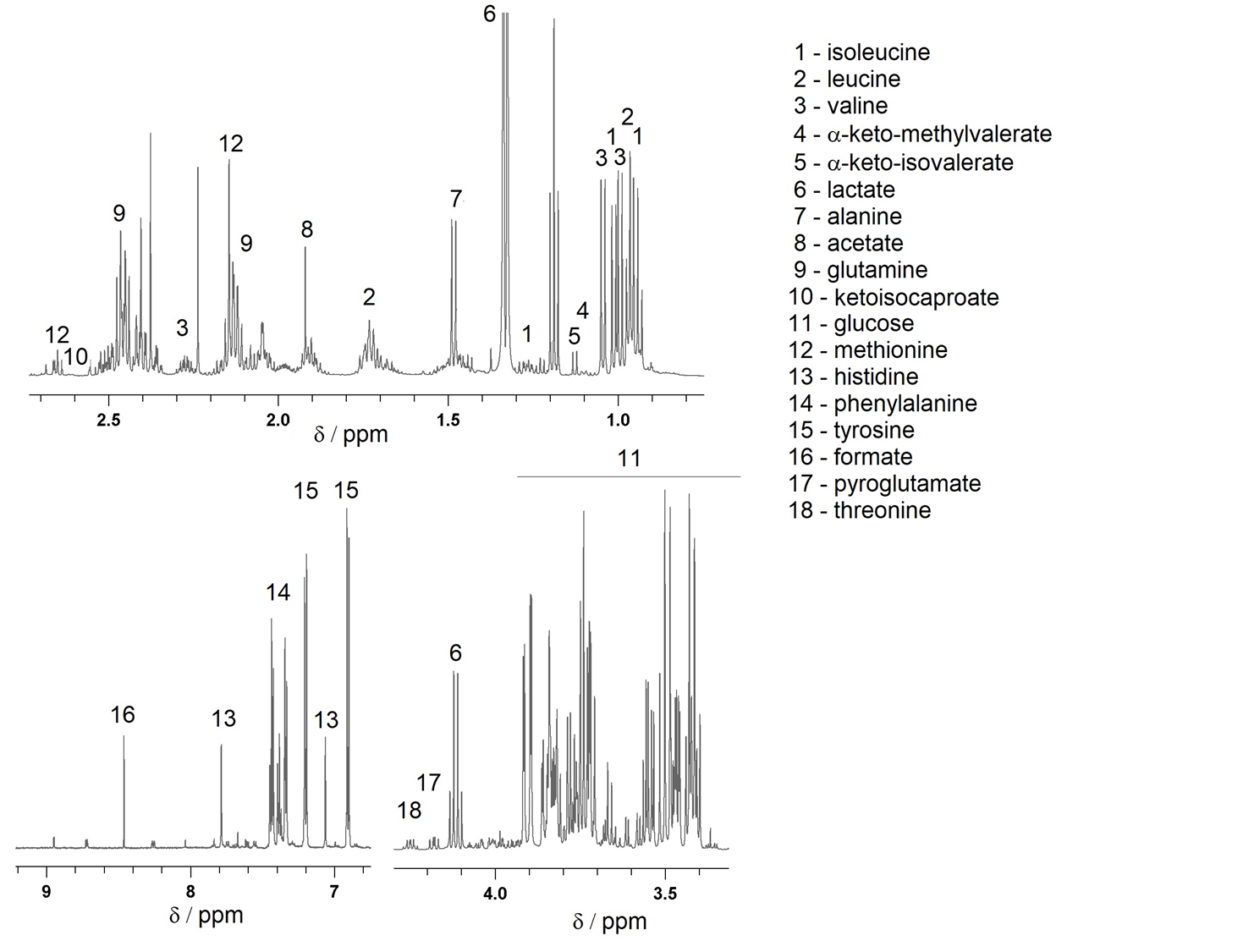


Figure S1.: Representative spectra of all analyzed metabolites of culture media consisting of DMEM/F12 supplemented with 10% FBS after 24 h incubation with neuroblastoma cells. All spectra were obtained by ^1^H-NMR spectroscopy and the ranges of chemical shifts (δ) for all mentioned metabolites are depicted.


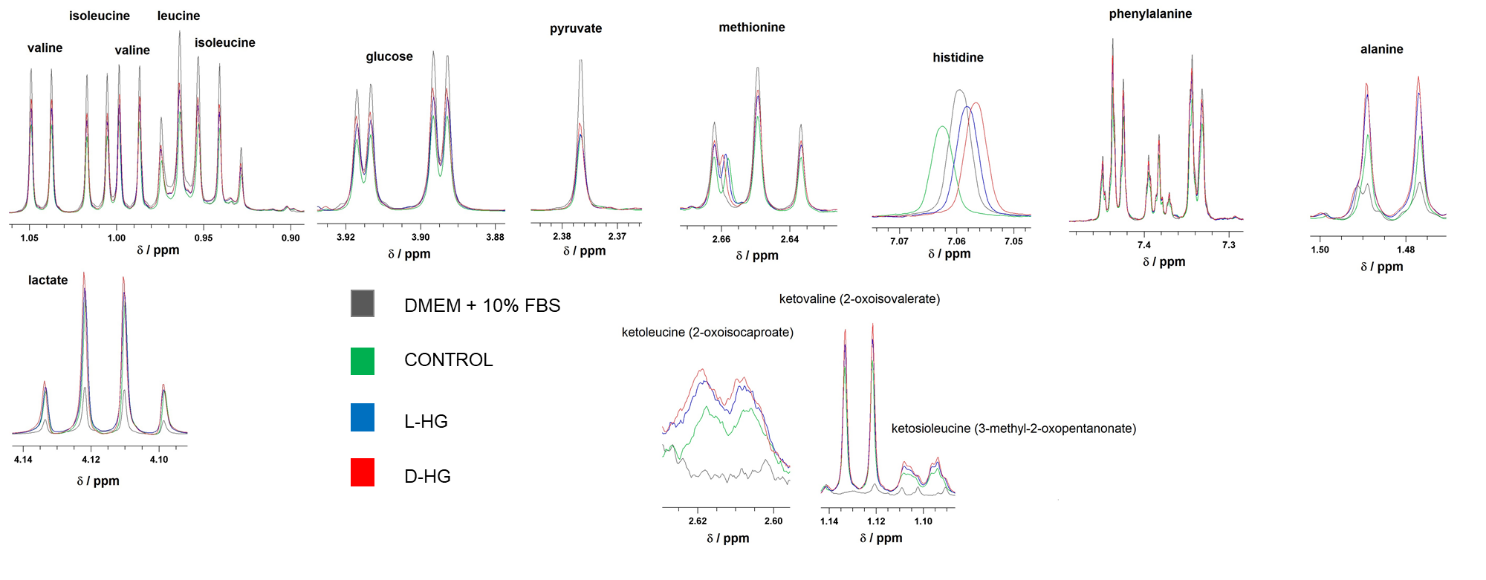


Figure S2.: Representative spectra of glucose; lactate; pyruvate; alanine; branched-chain amino acids, namely leucine, isoleucine, valine; branched-chain keto acids, namely α-ketoisocaproate, α-keto-methylvalerate, α-ketoisovalerate; and essential amino acids: methionine, histidine, and phenylalanine of culture media consisting of DMEM/F12 supplemented with 10% FBS without L or D-hydroxyglutarate (CONTROL, green), or with addition of 0.1 mM L-hydroxyglutarate (L-HG, blue), or with 0.1 mM D-hydroxyglutarate (D-HG, red) after 24 h incubation with neuroblastoma cells. Representative spectra of mentioned metabolites of culture media consisting of DMEM/F12 supplemented with 10% FBS without L or D-hydroxyglutarate before 24 h incubation (DMEM/F12 + 10% FBS, black) served as internal standard. All spectra were obtained by ^1^H-NMR spectroscopy and the ranges of chemical shifts (δ) for all mentioned metabolites are depicted.
